# Evaluation of deep learning models for segmentation of hippocampus volumes from MRI images in Alzheimer’s disease

**DOI:** 10.1038/s41598-026-38220-4

**Published:** 2026-02-09

**Authors:** Yori Pusparani, Chih-Yang Lin, Yih-Kuen Jan, Ben-Yi Liau, Fu-Yu Lin, Elvin Nur Furqon, Muhammad Talal, Sheena Christabel Pravin, Zhi-Ren Tsai, Chi-Wen Lung

**Affiliations:** 1 Department of Visual Communication Design, Budi Luhur University, Jakarta, 12260 Indonesia; 2 Department of Digital Media Design, Asia University, Taichung, 413305 Taiwan; 3 Department of Mechanical Engineering, National Central University, Taoyuan, 320317 Taiwan; 4 Rehabilitation Engineering Lab, Department of Health and Kinesiology, University of Illinois at Urbana-Champaign, Urbana, IL 61801 USA; 5 Department of Automatic Control Engineering, Feng Chia University, Taichung, 407102 Taiwan; 6 Department of Neurology, China Medical University Hospital, Taichung, 404327 Taiwan; 7 Department of Computer Engineering, Gachon University, Seongnam-si, 13120 South Korea; 8 School of Electronics Engineering, Vellore Institute of Technology, Chennai, 600127 India; 9 Department of Computer Science and Information Engineering, Asia University, Taichung, 413305 Taiwan; 10 Department of Medical Research, China Medical University Hospital, Taichung, 404327 Taiwan; 11 Department of Creative Product Design, Asia University, Taichung, 413305 Taiwan; 12 Center for Precision Medicine Research, Asia University, Taichung, 413305 Taiwan

**Keywords:** Hippocampus, U-Net, YOLO-v8, DeepLab-v3, Volume loss, Segmentation, Alzheimer's disease, Brain, Cognitive ageing, Learning algorithms, Alzheimer's disease, Alzheimer's disease

## Abstract

The hippocampus is a crucial brain structure associated with Alzheimer’s disease (AD). Precise segmentation is crucial for studying AD progression using deep learning. This study aimed to evaluate the performance of deep learning models in segmenting the left and right hippocampus in MRI images. We hypothesized that deep learning-based approaches would enable precise and accurate segmentation of the left and right hippocampus. We propose U-Net, You Only Look Once version 8 (YOLO-v8), and DeepLab-v3 models using the Alzheimer’s Disease Neuroimaging Initiative (ADNI) dataset. This study used 300 subjects, comprising 100 subjects (AD), 100 subjects with mild cognitive impairment (MCI), and 100 subjects with normal control (NC), resulting in a total of 7859 image slices. The results showed that the U-Net model exhibited the best Intersection over Union (IoU), which served as a key performance indicator among the three classes: AD (0.639), MCI (0.801), and NC (0.751). In contrast, YOLO-v8 demonstrated lower IoU performance for AD (0.342), MCI (0.465), and NC (0.550), which are considered inappropriate models to segment the left and right hippocampus. We obtained the left hippocampus volume of AD (1557.5 mm³), MCI (1863.3 mm^3^), and NC (2089.2 mm^3^). The right hippocampus volumes of AD (1593.4 mm³), MCI (1918.7 mm^3^), and NC (2280.2 mm^3^). The U-Net model exhibited the best performance. We expect deep learning-based methods to assist in clinical decisions by providing accurate hippocampus segmentation.

## Introduction

Alzheimer’s disease (AD) is the most common form of dementia. In 2021, 2 million deaths from AD and other dementias were reported worldwide^1^. According to the estimates of the Global Burden of Disease 2019 study, an increase in the number of persons with dementia from 57.4 million in 2019 to 152.8 million in 2050 will be observed globally. The primary etiological factors underlying AD require further investigation and validation^2^. It has consistent epidemiological trends and is a growing global health challenge. Early diagnosis offers potential benefits for patients, including timely access to medical treatments that can slow disease progression. The possibility of early diagnosis can be based on a specific biomarker^3^. Hence, identifying and investigating biomarkers is crucial, as such insights may facilitate the evaluation of disease progression and support the development of more effective treatment strategies.

In identifying biomarkers for early AD diagnosis, various potential biomarkers are available, including neuroimaging, cerebrospinal fluid biomarkers, blood biomarkers such as phosphorylated tau and amyloid-β 5, and Mini-Mental State Examination (MMSE) scores, which inform clinical decision-making and patient care. Indeed, blood biomarkers require clear preanalytical guidance on the effect of common covariates on the biomarker levels required, and the MMSE score may not be sensitive enough to detect subtle cognitive decline^4^. On the other hand, volumetric magnetic resonance imaging (MRI) of the hippocampus is the best established biomarker for AD^5,6^. This is because MRI has a high spatial resolution and is sensitive to changes in shape and volume within the brain. Thus, measuring the hippocampus volume with MRI-based assessments to observe changes has proven to be an effective predictor of progression and is more practical for clinical use in the early diagnosis of AD^7–9^.

To some extent, the hippocampus plays an important role as a biomarker of AD and is a valuable target for developing preventive therapies^10,11^. The hippocampus is located in the medial temporal lobe, comprising the left and right hippocampus on both sides of the brainstem, near the cerebellum. The hippocampus volume loss is closely associated with cognitive decline, which serves as a significant indicator of an elevated risk of AD progression. A previous study reported volume loss in the left and right hippocampus^12^. The left hippocampus volume may be associated with both episodic and working memory^13^. Spiers et al. demonstrated that left hippocampus volume loss was considered to be predictive of future dementia. The right hippocampus is predominantly associated with spatial navigation and topographical memory^14^. The left and right hippocampus volume loss is a risk factor for AD, and it has been suggested to affect the progressive phases of memory loss differentially^15^. However, monitoring the left-right hippocampus volumetric loss in MRI images enables clinicians to identify early signs of AD, allowing for timely interventions and targeted prevention strategies to slow or halt disease progression. The novelty of knowing the left and right hippocampus volumes can be used in the early stages to compare the pattern across disease progression.

The left and right hippocampus are relatively small regions of MRI images, which pose challenges for volume measurement and require accurate delineation in MRI images. Previous studies have encompassed various methods for measuring hippocampus volume^6^. First, manual segmentation, which requires an expert to trace the hippocampus slice-by-slice in MRI images, is typically performed using ITK-SNAP (http://www.itksnap.org/pmwiki/pmwiki.php), and the total volume is calculated by summing the segmented areas across slices and multiplying them by the slice thickness. Experts may choose slightly different boundaries for the hippocampus from MRI imaginges. It is the gold standard in clinical practice^16,17^. This method is time-consuming and requires extensive anatomical knowledge, and its results largely depend on the evaluator’s experience^18^. Additionally, it may limit the feasibility of volumetric quantification of large datasets. An automated method to segment the hippocampus is available, such as FreeSurfer. This is one of the most widely used methods for hippocampus segmentation, in which MRI images are fed into software to obtain the volume automatically^19–21^. Moreover, it is fairly reliable in delineating the volumes of an easy-to-segment structure, such as the caudate, but not the hippocampus, despite being scalable for large datasets^22^. Recently, an expansion of deep learning in medical imaging has been proposed to optimize hippocampus segmentation, which is faster^23^.

With technological advancements, the use of deep learning models has proven to be beneficial for various tasks. For example, the use of Convolutional Neural Networks (CNNs) for fault diagnosis offers an effective solution for real-time system prediction and monitoring^24,25^. In contrast, a study was conducted on medical images, such as the classification of breast cancer using explainable AI^26^, and burn tissue segmentation using bidirectional long short-term memory (Bi-LSTM)^27^, which was used to reduce the computational time. Indeed, deep learning based methods have achieved impressive results in many medical imaging segmentation tasks^21,28^. It generally produces segmentations that are more accurate than traditional automatic methods in a fraction of the time^23^. Segmenting small regions in MRI images, such as the hippocampus, is challenging. For example, in the lesion area, the small pixel size of the dataset makes segmentation impractical, which means that the model fails to accurately identify the lesion area^29^. Several techniques have been proposed to improve deep learning performance. Techniques such as Markov random fields (i.e., undirected graphical models) have been used to improve the segmentation performance in small regions. This is a post-processing step, and post-processing is not part of the training of the segmentation network^30^. It is applied to the dataset after training; the network cannot adapt its weights based on the post-processing. On the other hand, data augmentation has been proposed to enhance segmentation accuracy, owing to the increasing diversity of training examples, which helps models capture subtle anatomical variations more effectively^31^. Notably, the challenge of applying deep learning to segment the left and right hippocampus in MRI images may be mitigated through data augmentation, which could potentially enhance the model’s performance. The main contribution is that the deep-learning approaches have not been studied to validate their use in segmenting the left and right hippocampus, which can provide a reliable basis for estimating hippocampus volume loss in clinical studies.

### Hypothesis and purpose

**Fig. 1:**
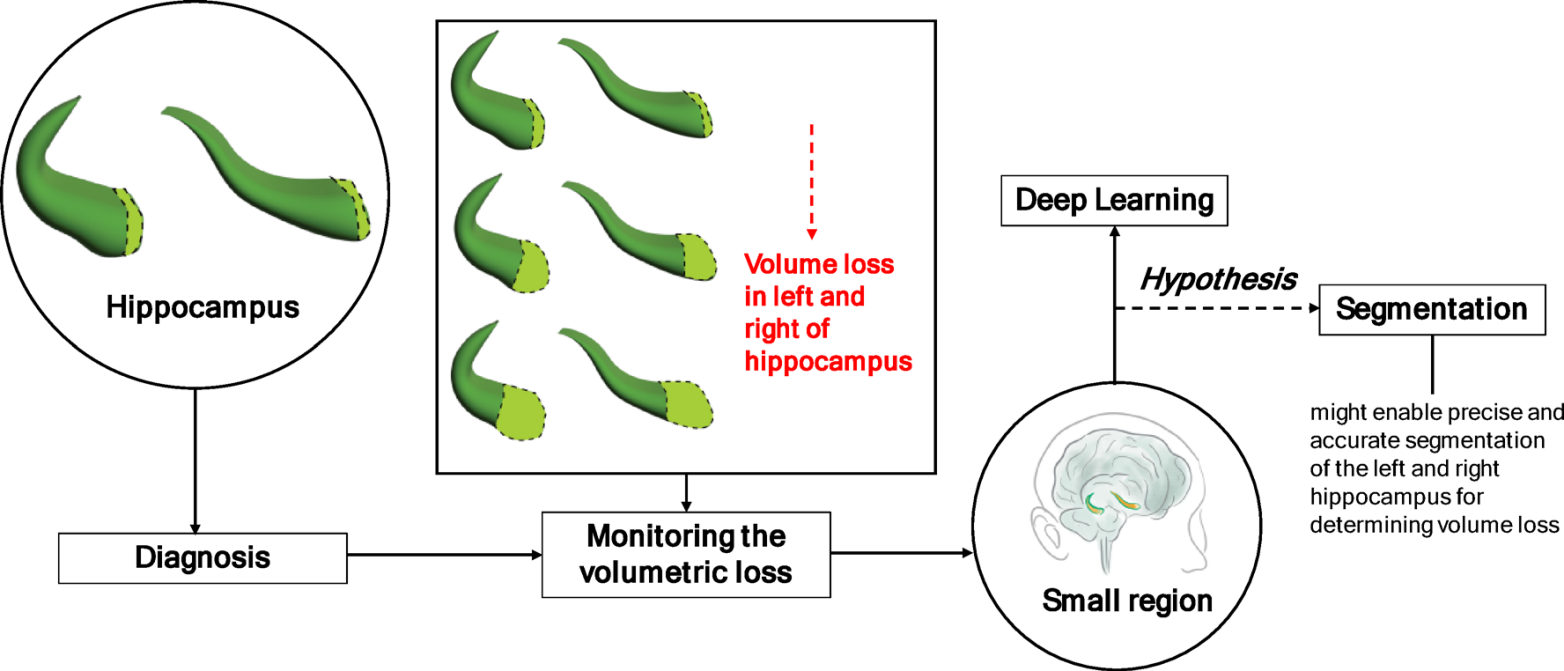
The diagram provides a schematic representation of the hypothesis.

According to Fig. 1, first, in AD, the hippocampus is a biomarkers that play a crucial role in early diagnosis. The hippocampus is particularly important for the diagnosis of AD. Second, monitoring volumetric loss provides critical insights into disease progression in both the left and right hippocampus. Hippocampus volume changes are associated with cognitive functions affected by AD. However, the hippocampus is a relatively small region of the brain, making it challenging to determine volume loss accurately. Third, with the development of technology, deep learning is essential for segmenting small regions of the brain. We hypothesized that deep learning-based approaches might enable precise and accurate segmentation of the left and right hippocampus to determine volume loss.

This study aimed to evaluate the performance of deep learning models in segmenting the left and right hippocampus in MRI images. We measured the hippocamus volume in the three classes. The use of three classes could enhance the interpretability of disease progression in the left and right hippocampus. Thus, it may provide valuable insights into disease progression, facilitating early intervention, enabling medical professionals to diagnose hippocampus volume loss more effectively.

## Result

### Comparison of each model

**Fig. 2:**
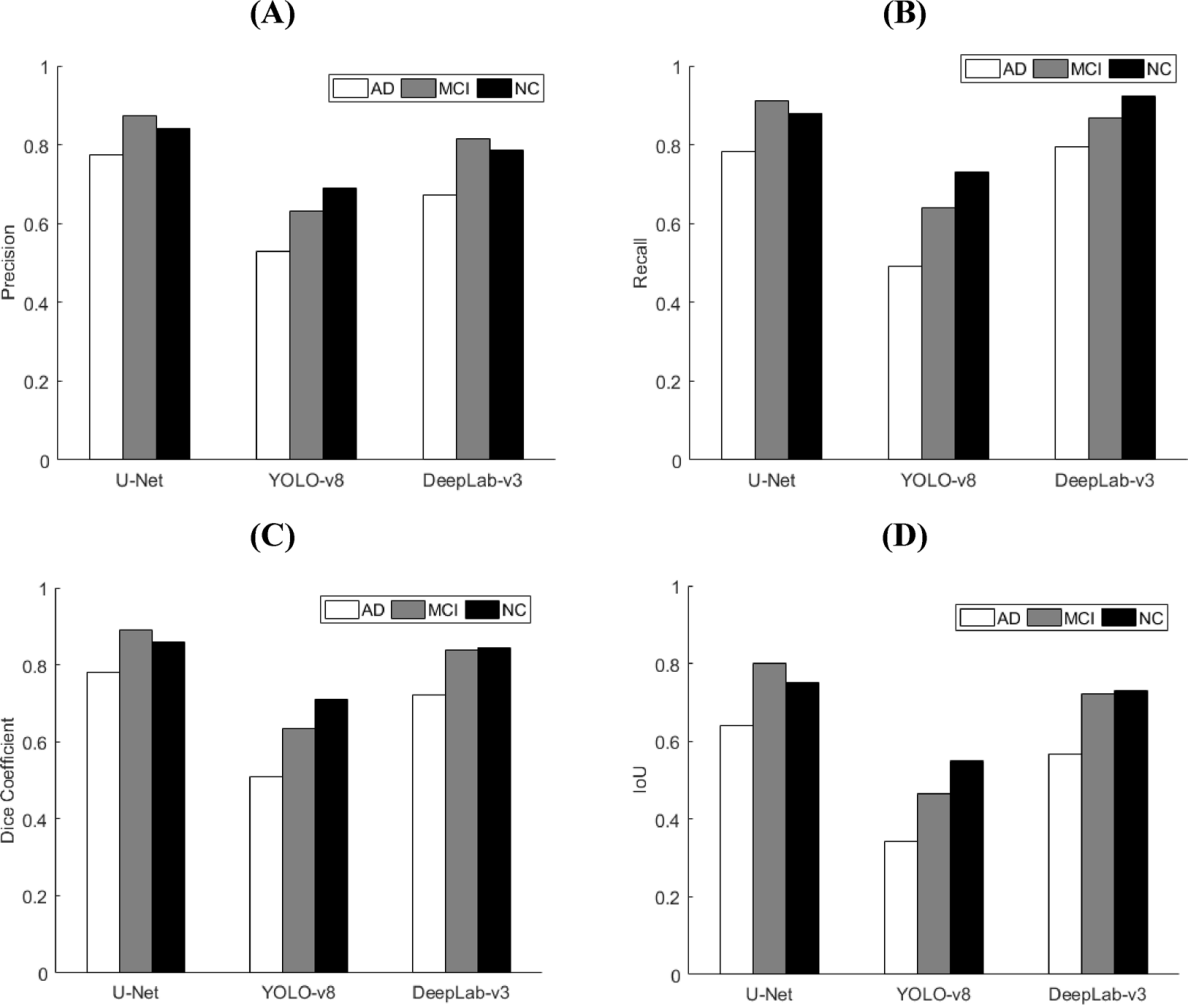
The metric performance of the proposed models in three classes: (**a**) Precision; (**b**) Recall; (**c**) Dice coefficient; (**d**) IoU. AD, Alzheimer’s disease; MCI, mild cognitive impairment; NC, normal control; IoU, intersection over union.

We compared the three models in the three classes to determine the best performance in segmenting the hippocampus. We quantified the comparison using four metrics: loss, precision, recall, Dice coefficient, and IoU. However, the IoU results showed that the U-Net model performed best in segmenting the left and right hippocampus for AD (0.639), MCI (0.801), and NC (0.751), respectively. In contrast, the YOLO-v8 model obtained lower IoU results than the other models. Table 1; Fig. 2 show the detailed performance of the proposed model for the three classes.

**Table 1:** The metric performance of the proposed model in the three classes.

Metric performance	U-Net			YOLO-v8			DeepLab-v3		
	AD	MCI	NC	AD	MCI	NC	AD	MCI	NC
Precision	0.775	0.872	0.842	0.530	0.630	0.690	0.672	0.814	0.785
Recall	0.784	0.910	0.880	0.490	0.640	0.730	0.794	0.866	0.924
Dice coefficient	0.780	0.890	0.858	0.509	0.635	0.709	0.723	0.839	0.845
IoU	0.639	0.801	0.751	0.342	0.465	0.550	0.567	0.722	0.731

*Note: AD,* Alzheimer’s disease; *MCI,* mild cognitive impairment; *NC,* normal control; *IoU,* Intersection over Union.

## K-fold cross-validation

The three-fold cross-validation yielded an average IoU performance of 0.71 (AD), 0.71 (MCI), and 0.72 (NC). For each cross-validation fold, the training and prediction scores are summarized in Table 2.

**Table 2:** K-fold cross-validation performance in three classes.

K-fold	Precision			Recall			Dice coefficient			IoU		
	AD	MCI	NC	AD	MCI	NC	AD	MCI	NC	AD	MCI	NC
Fold 1	0.76	0.82	0.83	0.87	0.87	0.90	0.81	0.85	0.87	0.68	0.74	0.77
Fold 2	0.83	0.81	0.74	0.87	0.82	0.88	0.85	0.81	0.80	0.74	0.69	0.67
Fold 3	0.79	0.80	0.85	0.88	0.86	0.83	0.83	0.83	0.84	0.71	0.71	0.73
Average	**0.79**	**0.81**	**0.81**	**0.87**	**0.85**	**0.87**	**0.83**	**0.83**	**0.84**	**0.71**	**0.71**	**0.72**

Note 1: Significant values are in bold.

*Note 2: AD*, Alzheimer’s disease; *MCI,* mild cognitive impairment; *NC,* normal control; *IoU,* Intersection over Union.

### Hippocampus volume in the testing dataset

**Fig. 3:**
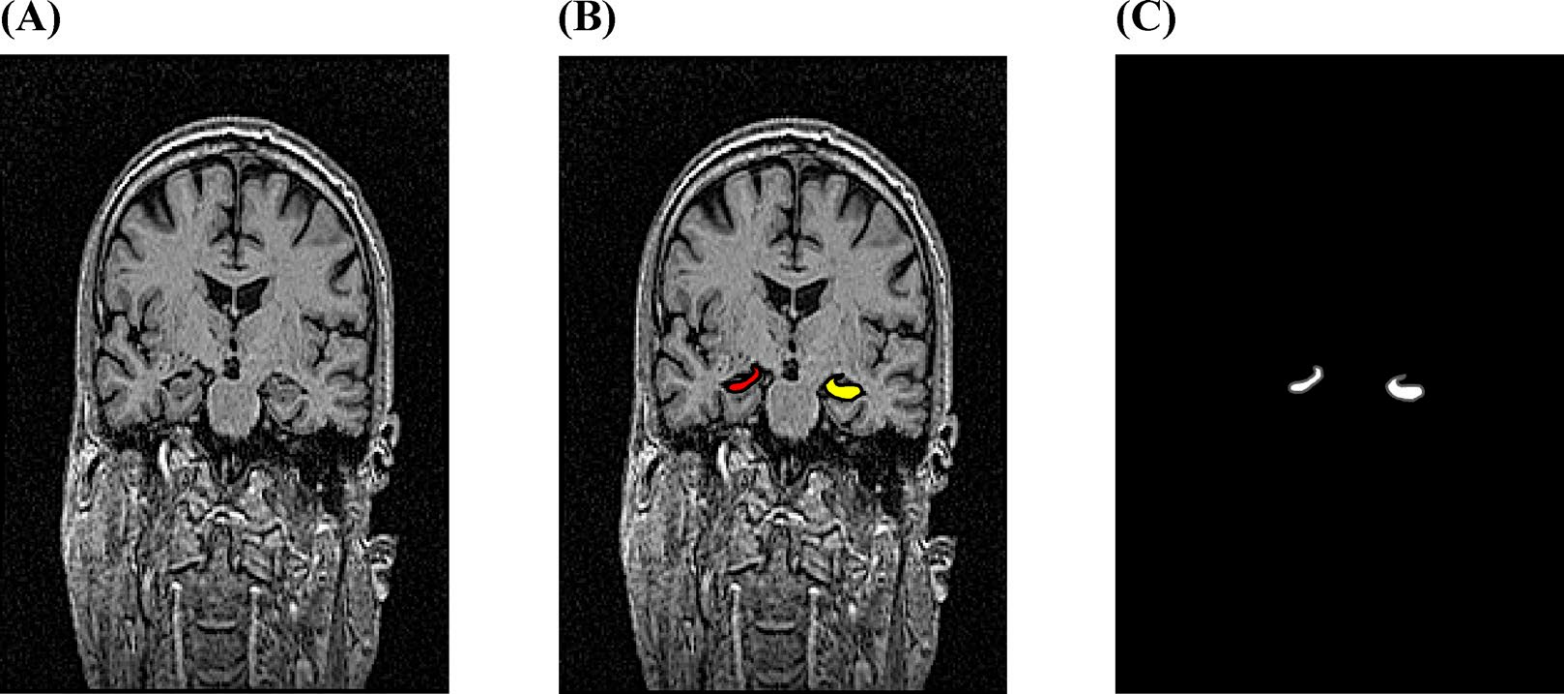
The left and right hippocampus segmentation from the sample MRI images sourced from the Alzheimer’s Disease Neuroimaging Initiative (ADNI) dataset; (**a**) The input images; (**b**) The prediction of segmentation images; and (**c**) The hippocampus segmented area.

Furthermore, the U-Net model was selected, which demonstrated the best IoU performance across the three classes (i.e., AD, MCI, and NC), and was used to predict segmentation masks for volume estimation using a testing dataset. The hippocampus segmentation prediction results are presented in Fig. 3.

**Fig. 4:**
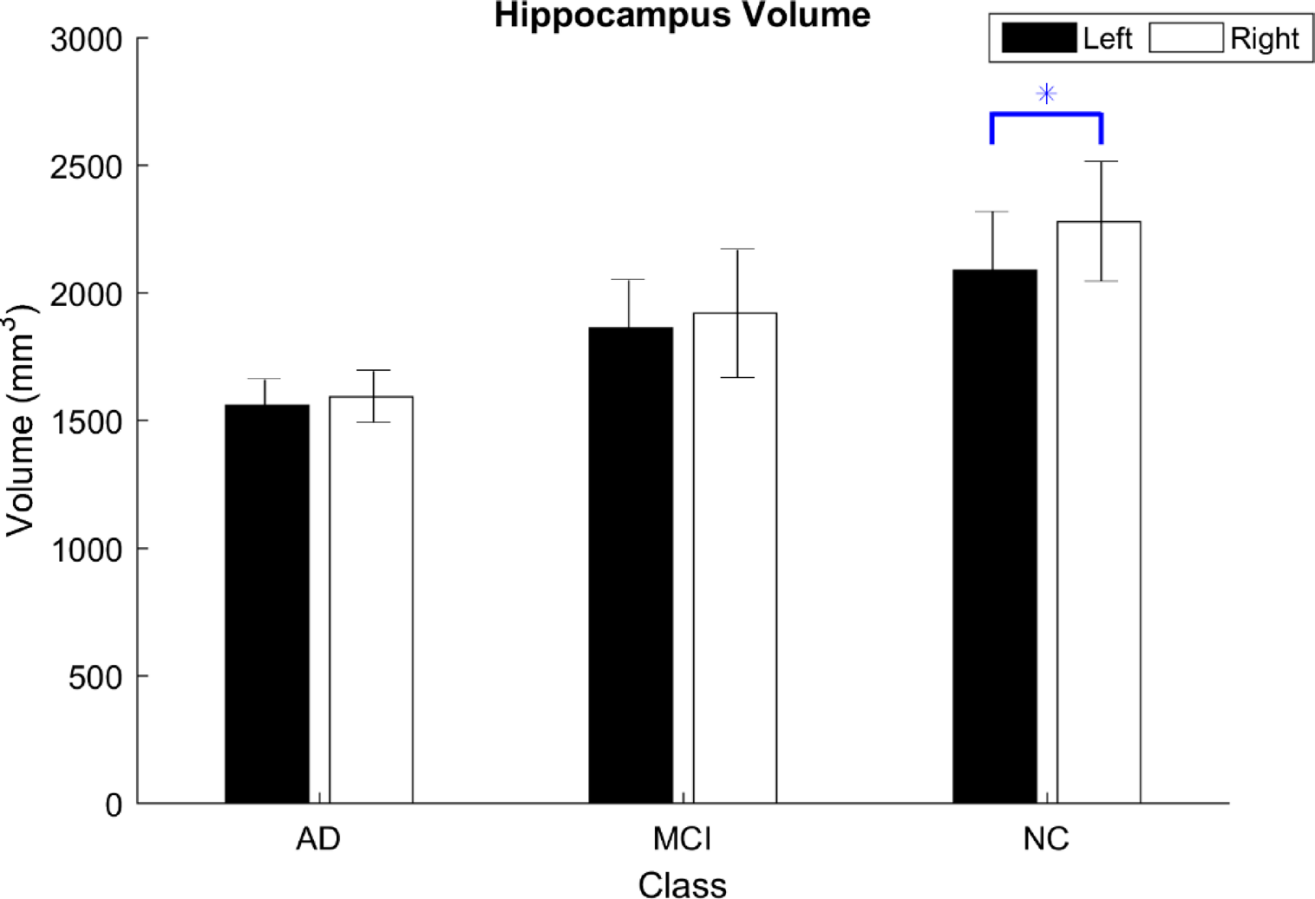
The left and right hippocampus volumes were classified into three classes. Note: AD, Alzheimer’s disease; MCI, mild cognitive impairment; NC, normal control; SD, standard deviation. (∗*p* < 0.05; values are the mean with standard deviation).

As presented in Table 2, hippocampus volume was smaller on the left side across the three classes (i.e., AD, MCI, and NC), with values of 1558 mm³, 1863 mm³, and 2,089 mm³ for AD, MCI, and NC, respectively. Furthermore, we calculated the asymmetry index of the left and right hippocampus volumes for the three classes. Table 3 presents the results for the three classes, with an asymmetry index greater than zero, indicating that the right hippocampus was larger than the left. In addition, we performed a paired *t* test to compare the left and right hippocampus volumes within each of the three classes. It was used to identify which side was more affected and the class of disease progression. We found significant differences between the left and right hippocampus in the NC group (*p* = 0.043), as shown in Table 4; Fig. 4.

**Table 3:** The hippocampus volume of the three classes.

Subject	AD (subject 1–10)			MCI (subject 11–20)			NC (subject 21–30)		
	Left (mm^3^)	Right (mm^3^)	Left-right asymmetry index (%)	Left (mm^3^)	Right (mm^3^)	Left-right asymmetry index (%)	Left (mm^3^)	Right (mm^3^)	Left-right asymmetry index (%)
Subject 1	1555	1646	2.8	1846	2000	4.0	2133	2335	4.5
Subject 2	1497	1365	− 4.6	1843	1949	2.8	1899	2287	9.3
Subject 3	1387	1593	6.9	2113	1746	− 9.5	2182	1974	− 5.0
Subject 4	1557	1663	3.3	1877	1825	− 1.4	2417	2353	− 1.3
Subject 5	1455	1621	5.4	1633	2295	16.9	2133	2335	4.5
Subject 6	1670	1553	− 3.6	1633	2295	16.9	1889	1968	2.0
Subject 7	1725	1695	− 0.9	2071	1730	− 9.0	1633	2295	16.9
Subject 8	1625	1497	− 4.1	1625	1533	− 2.9	2050	2031	− 0.5
Subject 9	1603	1697	2.8	2063	1744	− 8.4	2260	2536	5.8
Subject 10	1501	1604	3.3	1929	2070	3.5	2296	2688	7.9
Average	**1** **557.5**	**1** **593.4**	**1.1**	**1** **863.3**	**1** **918.7**	**1.3**	**2** **089.2**	**2** **280.2**	**4.4**

Note 1: Significant values are in bold.

*Note 2: AD*, Alzheimer’s disease; *MCI,* mild cognitive impairment; *NC,* normal control.

**Table 4:** A paired *t* test of the left and right hippocampus volume in three classes.

Class (*N*)	Hippocampus volume (mm^3^)		Paired *t* test
	Left	Right	
	(Mean ± SD)	(Mean ± SD)	
AD (10)	1557.5 ± 102.1	1593.4 ± 101.5	0.394
MCI (10)	1863.3 ± 186.6	1918.7 ± 251.0	0.651
NC (10)	2089.2 ± 230.4	2280.2 ± 234.9	0.043*

Note 1: Significant values are in bold.

*Note 2: AD,* Alzheimer’s disease; *MCI,* mild cognitive impairment; *NC,* normal control; *SD,* standard deviation. (∗, *p* < 0.05; values are mean ± standard deviation).

## Discussion

The results are consistent with our hypothesis that deep learning-based approaches will enable precise and accurate segmentation of the left and right hippocampus. This study demonstrated that the U-Net model performed better than the YOLO-v8 and DeepLab-v3 models. Previous studies have demonstrated that the U-Net architecture, characterized by its U-shaped design, effectively addresses numerous challenges in segmenting the hippocampus^28^. In this study, we further validated that the U-Net model approach can be used to obtain a small region of the hippocampus. Thus, segmenting the left and right hippocampus is crucial before volume measurements. Furthermore, the left hippocampus was significantly smaller than the right hippocampus in the NC class.

According to Table 1, we demonstrate that the U-Net model performs better in segmenting the hippocampus than the other two models. Indeed, the U-Net model has proven to be a successful method for segmentation, particularly when applied to small regions in medical images. This is consistent with the results of a previous study^28^. Therefore, the U-Net model has long been a benchmark architecture for traditional medical image segmentation tasks, particularly in clinical applications^32,33^. Several findings demonstrated that the two main methods can contribute to the U-Net model, which performed the best. First, applying a filtered image was demonstrated as a preprocessing step to enhance the training performance^34^. Second, the most well-known method is the use of data augmentation to balance the sample size of the dataset^31^. We confirmed that the above two methods improved the U-Net. Nevertheless, the potential underlying reasons may involve multiple contributing methods that vary depending on the specific purposes and applications. Therefore, our findings suggest that the U-Net model can be regarded as a reliable method for hippocampus segmentation, particularly in small regions of medical images. In addition, K-fold cross-validation (see Table 2) was used to ensure that each validation set remained reasonably sized, which provided robust estimates of the model performance. This may imply that a K-fold was chosen to preserve the class distributions and ensure stable performance measurement across folds. Previous studies have employed several K-fold methods for various models and datasets, achieving an average IoU performance range of 0.73 to 0.84, with similar or even slightly lower parameter requirements^35^. We selected the IoU metrics performance since it directly reflects the overlap quality between predicted and ground truth segmentations, which is critical for estimating the volume. Even though we used the same parameters, such as epoch and batch size, for segmenting the left and right hippocampus, the U-Net still demonstrated the best performance. Even though YOLO-v8 can balance the computational efficiency and high-quality segmentation for some cases, such as remote sensing images^36^, despite the U-Net being a strong baseline for segmentation^33^.

In addition, the results demonstrated that the YOLO-v8 model exhibited lower IoU scores in segmenting the left and right hippocampus across three classes in AD (0.342), MCI (0.465), and NC (0.550) compared to other proposed models. These limitations can be attributed to the constrained capacity of the model to capture fine-grained structural details in small or irregular regions. These findings suggest that YOLO-v8 may be inadequate for accurately delineating and localizing the hippocampus, particularly for simultaneously identifying and refining anatomical boundaries. A previous study also employed YOLO-v8 for brain tumor segmentation, where IoU scores were reported to be below 0.30^37^. Their studies suggest that the model should consider utilizing the backbone, which can be applied in medical imaging scenarios, to enhance the YOLO-v8 model’s capabilities.

To some extent, a previous study found that using the YOLO-v8 model for lung lesion segmentation has promising results using computed tomography (CT) images. The model achieved an IoU score of 0.821, which was improved using regularization strategies. In addition, future research should focus on multi-dataset training to enhance generalization, using the YOLO-v8 model to handle diverse image types^38^. Thus, we may say that using the YOLO-v8 model to segment the hippocampus is still an emerging field and requires further exploration. Indeed, the YOLO-v8 model may not be well-suited for segmentation tasks, particularly when dealing with small regions in MRI images.

**Fig. 5:**
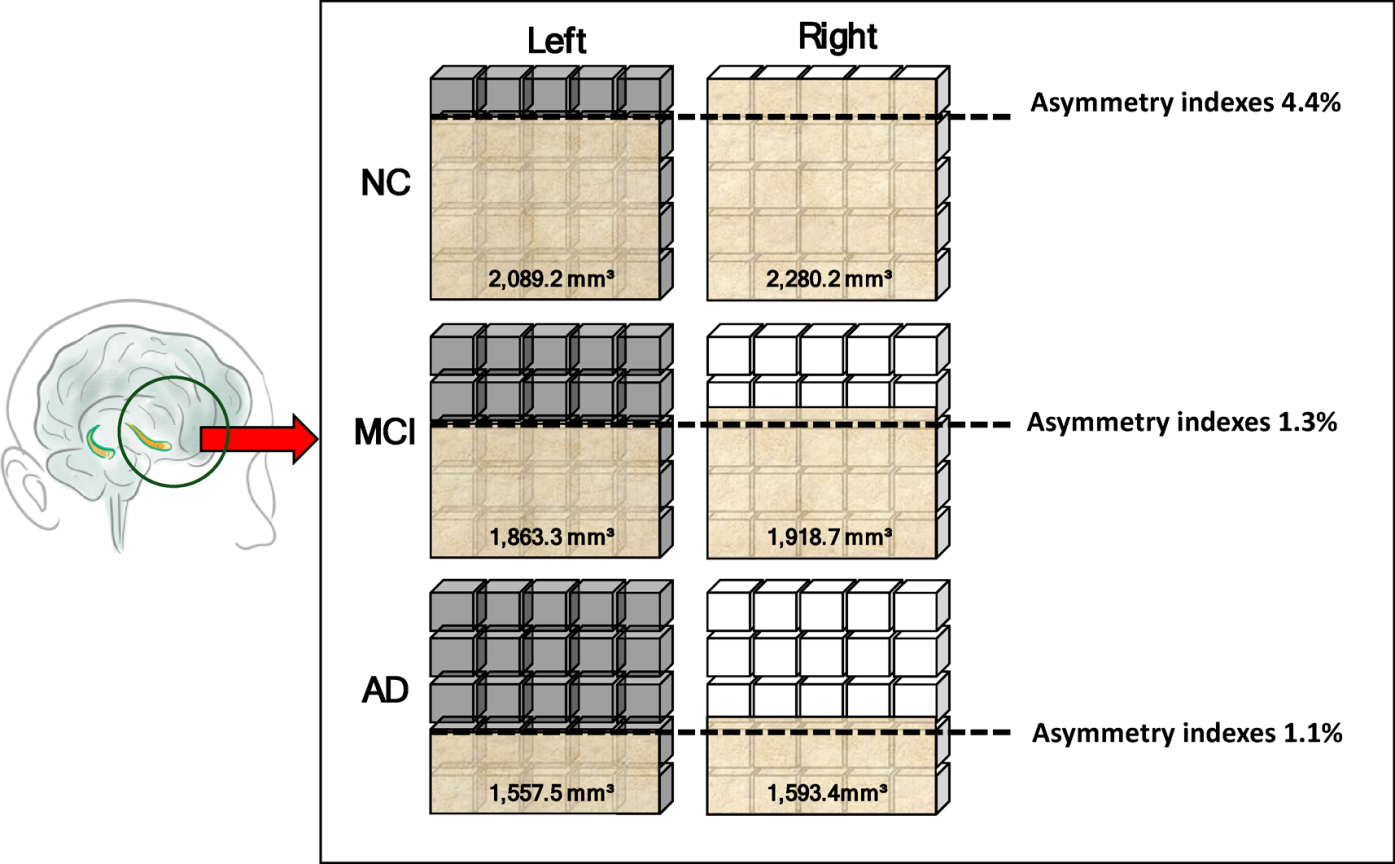
The illustration shows the volume asymmetry of the left and right hippocampus in the three classes.

Additionally, hippocampus volume measurement was performed using a deep learning approach. Table 3 indicates that the left hippocampus volume was smaller among the three classes. By comparing the left and right hippocampus volumes, a consistent left-less-than-right asymmetry pattern was found, with asymmetry index scores of 1.1% (AD), 1.3% (MCI), and 4.4% (NC), indicating that the highest asymmetry index was found in the NC class (Fig. 5). In contrast, previous studies have shown asymmetry index scores of 5.07% (AD), 2.99% (MCI), and 1.91% (NC). We may say that the reasons for these different score results may originate from differences in the segmentation method or the reliability of the MRI images^39^. Moreover, the consistency of the right was bigger than that of the left, which aligns with previous meta-analyses^15,40^.

To some extent, the average volume reduction by sample size was 12.9% and 11.1% in the left and right hippocampus in MCI and 24.2% and 23.1% in the left and right hippocampus, respectively, in AD^15^. Indeed, the left hippocampus volume loss was associated with more severe cognitive problems than the right^41^. The left hippocampus contributes to long-term memory, whereas the right hippocampus contributes to short-term memory. Previous research suggests that damage to the left hippocampus often results in deficits in verbal memory, characterized by difficulties in remembering words, lists, or stories. In contrast, damage to the right hippocampus leads to issues in spatial memory, such as getting lost or forgetting locations. Specifically, the left hippocampus is primarily responsible for context-dependent episodic or autobiographical memory, whereas the right hippocampus is involved in the memory of locations within the environment. Episodic memory is the first symptom experienced by patients with AD with medial temporal lobe damage due to progressive pathology. To prevent this, a study found that modern neurostimulation approaches effectively activate the hippocampus to enhance episodic memory performance^42^. In contrast, the functional connectivity of the right hippocampus has been associated with the efficacy of cholinesterase inhibitors, a common AD treatment^43^. Thus, understanding how hippocampus volume loss on both sides benefits researchers in detecting, localizing, and diagnosing patients with AD. Nevertheless, this finding could lead to the early and accurate identification of conditions, allowing for enhanced medical care and timely patient treatment.

Additionally, the asymmetry index may have some potential reasons, such as image orientation^44^. The studies found that a bias could occur on the side of the screen on which the hippocampus was viewed, such that hippocampus volume was larger when traced on the left side of the screen than when traced on the right. Thus, a bias may exist that needs to be avoided in future studies.

This study had several limitations. First, we proposed only three models to segment the left and right hippocampus, which may have biased the conclusion. In future studies, for a broader exploration of deep learning performance and to avoid bias, another deep learning model, such as a multitask deep CNN model^21^, can be proposed to compare the performance in segmenting the left and right hippocampus. This model was designed to learn multiple related tasks simultaneously. However, we may consider applying a method with a recent hybrid model for feature extraction as an alternative to combining data sources to obtain a deeper understanding of the features. Previous studies have been conducted in a fault-diagnosis framework to ensure noisy signals, limited label data, or data collection^45^ or to apply dual-input deep learning, which can be beneficial for validating real data^46^.

Second, although the U-Net model demonstrated the highest Intersection over Union (IoU) performance in segmenting the left and right hippocampus from MRI images, its architecture has known limitations in capturing complex anatomical boundaries, particularly in small or morphologically variable structures, such as the hippocampus. Although the application of image processing and data augmentation improved model generalization, the base U-Net architecture may not fully leverage multi-scale contextual information or semantic gap reduction between encoder and decoder features. In future research, we may propose deep learning models by using U-Net +  + as another modification of the U-Net architecture to optimize the deep learning models^47^, as previous studies have found that the architecture of U-Net +  + showed deeper and more connections and captured fine details, which led to improved segmentation in small brain regions of medical images^48^. Hence, we assumed that it may be appropriate to enhance the performance in segmenting the left and right hippocampus, which are considered to be small regions.

Third, we used a dataset of 10 subjects to validate the U-Net model’s performance, which is considered a small dataset, for estimating hippocampus volume and evaluating the model’s performance. However, the small dataset may influence the robustness of our findings related to hippocampus volume estimation. However, it still aligns with a previous study, which used five subjects for the segmentation of hippocampus subfields^49^. In future studies, we will apply more datasets to our models. Previous studies have suggested that larger datasets affect deep learning performance^50^.

## Conclusion

In conclusion, compared with YOLO-v8 and DeepLab-v3, the U-Net model demonstrated comparatively better performance in segmenting the left and right hippocampus from MRI images among the three classes. These results indicate that the U-Net model may be a suitable approach for estimating hippocampus volume on both sides. In addition, our findings support the hypothesis that the deep learning model can segment the left and right hippocampus. We found that the volume analysis across the three classes (i.e., AD, MCI, and NC) revealed a consistent trend in which the left hippocampus tended to have a smaller volume than the right hippocampus. The asymmetrical index of the NC class was shown to be the bigger value, while in MCI and AD, it showed a smaller value. This change may serve as a potential biomarker for disease progression. Additionally, from the asymmetry index result, we found that the asymmetry index in the AD and MCI classes showed slight differences in the left and right hippocampus, compared to the NC class.

## Materials and methods

### Dataset collection

The data used in this study were sourced from the publicly accessible Alzheimer’s Disease Neuroimaging Initiative (ADNI) database, specifically from the first phase (ADNI-1). Baseline ADNI-1 imaging data, acquired using a 1.5T Tesla scanner, were analyzed after preprocessing with Magnetization Prepared Rapid Gradient Echo (MP-RAGE) and reconstructed at a 208 × 240 × 256 mm resolution. We used a coronal view with a slice thickness of 1.3 mm and a scan time of approximately 45 min per session. Detailed information is available on the ADNI website (https://adni.loni.usc.edu). This study employed the coronal view and included 300 subjects divided into three classes: AD (100 subjects), mild cognitive impairment (MCI) (100 subjects), and normal control (NC) (100 subjects). The subjects were male and female; detailed information on the dataset is presented in Table 5.

**Table 5:** Demographic information from the ADNI was used in our study.

Number of subjects	AD	MCI	NC
	100	100	100
Ranging age (years)	55 to 85	58 to 89	62 to 90
Male/female	46/54	69/31	57/43
Age (mean ± SD)	74.5 ± 7.3	75.3 ± 6.9	76.4 ± 5.5

*AD* Alzheimer’s disease, *MCI* mild cognitive impairment, *NC* normal control, *SD* standard deviation

Furthermore, MRI images in approximately 25–30 image slices in the coronal view were selected, covering the entire length of the hippocampus, because that area contains the most essential information. The method of selecting slices has the advantage of enhancing the performance of the deep learning model compared to using all slices^51^. The target datasets were split into training, validation, and test sets at a ratio of 80:10:10. The training set consisted of 5,850 image slices (80 subjects, 80%). In contrast, the validation and testing sets comprised 1195 (10 subjects, 10%) and 814 (10 subjects, 10%) image slices. The total number of datasets contained 7859 image slices. The dataset of the MRI images is presented in Table 6.

**Table 6:** Dataset description.

Class (subject)	Image slices			Total image slices
	Training (subject)	Validation (subject)	Testing (subject)	
AD (100)	1,884 (80)	471 (10)	275 (10)	2,630
MCI (100)	1,924 (80)	480 (10)	280 (10)	2,684
NC (100)	2,042 (80)	244 (10)	259 (10)	2,545
Summary	**5**,**850 (240)**	**1**,**195 (30)**	**814 (30)**	**7**,**859**

Note 1: Significant values are in bold.

*Note 2: AD,* Alzheimer’s disease; *MCI,* mild cognitive impairment; *NC,* normal control.

### Labeling images

**Fig. 6:**
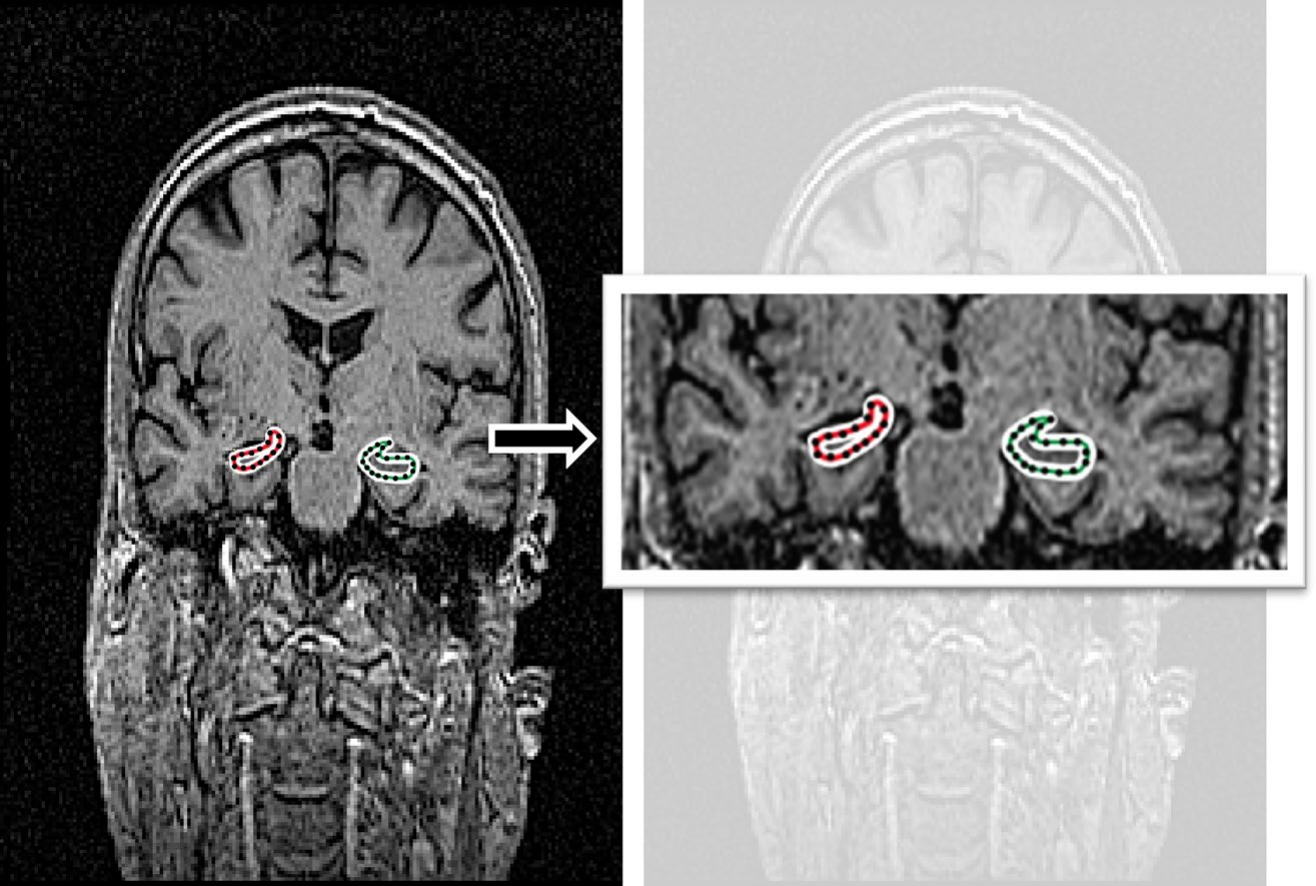
Exemplary labeling images in the AD class with sample MRI images sourced from the Alzheimer’s Disease Neuroimaging Initiative (ADNI) dataset.

Manual ground-truth labeling was performed in the coronal view. We labeled the MRI images that contained hippocampus structures on both the left and right sides. The left and right hippocampus structures in the training and validation sets were labeled for segmentation using labeling software (https://github.com/wkentaro/labelme). An experienced neurologist from China Medical University Hospital, who had worked in the field for over 15 years, reviewed the manual labeling process. The labeled images in the dataset are shown in Fig. 6.

### Image preprocessing and data augmentation

**Fig. 7:**
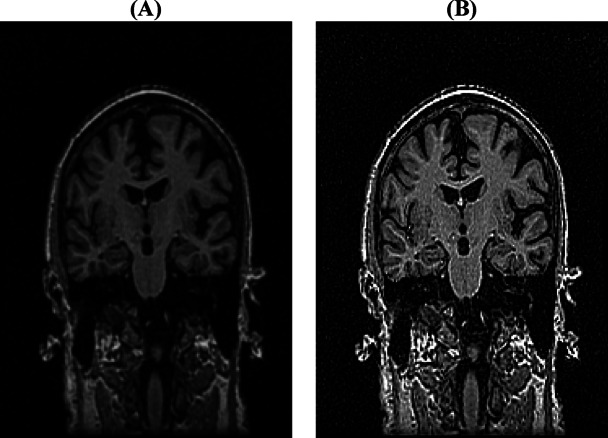
The preprocessed image dataset from the sample MRI images sourced from the Alzheimer’s disease neuroimaging initiative (ADNI) dataset: **(a)** Input image; **(b)** Filtered image.

We proposed preprocessing images with a Gaussian blur and image sharpening on our image dataset to enhance deep learning performance. It can also improve visual quality and emphasize the left and right hippocampus in the AD, MCI, and NC classes 34 (Eq. 1). The dataset obtained after image preprocessing is illustrated in Fig. 7.

1 $$G\left(x,y\right)=\frac{1}{{2\pi\:\sigma\:}^{2}}\:exp-\left(\frac{{x}^{2}+{y}^{2}}{{2\sigma\:}^{2}}\right)$$

where (*x*,* y*) is the coordinate of the kernel, $\sigma$ is the standard deviation of the Gaussian distribution (determines the ‘spread’ of the blur with = 0.5), and *exp* is the exponential function.

### Deep learning model

Medical image processing requires specialized deep learning techniques to determine the reliability of the left and right hippocampus segmentation in MRI images. We proposed the deep learning model, first the U-Net model, as it is the most well-known segmentation method and represents the most efficient architecture^32^. Second, the DeepLab-v3, which showed equivalent results in segmenting brain tumors using MRI images^52^. Third, a version of YOLO-v8 can also be used to perform segmentation^53^. This is because YOLO-v8 includes a mask prediction branch that enables instance-level segmentation, providing polygonal masks rather than just boxes, which has been done using YOLO-v8 for segmenting coronary artery disease from angiograms^54^. Therefore, previous studies have applied the U-Net model for hippocampus segmentation, but did not assess its performance in volume estimation^28^. Thus, with the advancement and comparable performance of deep learning models, we proposed U-Net, YOLO-v8, and DeepLab-v3 models to segment the left and right hippocampus. Additionally, the reliability of the proposed model can be examined by predicting and measuring hippocampus volume.

### U-Net

**Fig. 8:**
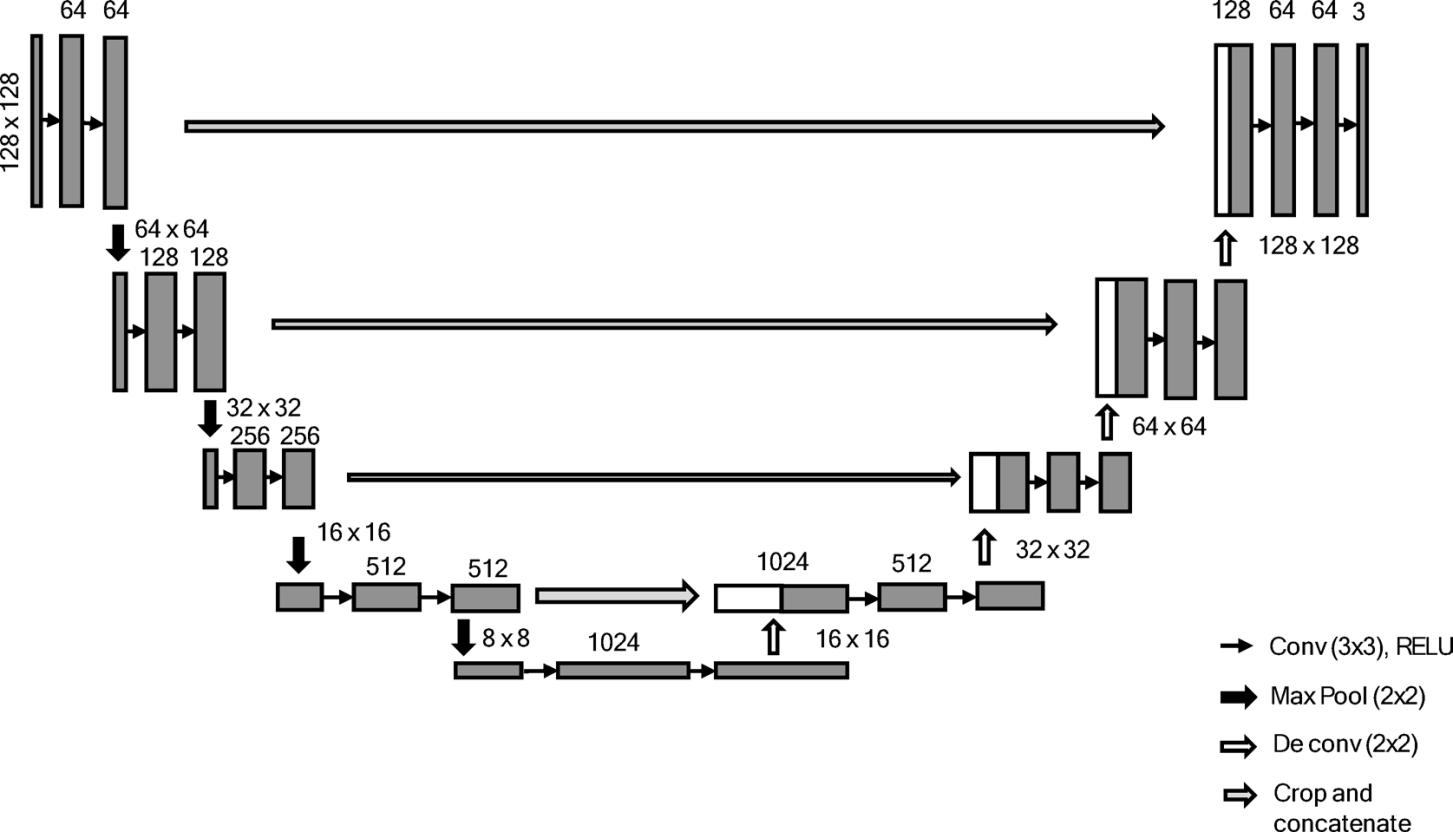
The U-Net architecture.

The U-Net architecture was introduced for biomedical image segmentation to enhance the robustness of the segmentation process^33^. The model architecture comprises two main components: a contracting path (encoder) and an expanding path (decoder). The encoder captures the context using repeated blocks of two 3 × 3 convolutions, ReLU activations, and a max-pooling layer to expand the receptive field and reduce computation. The decoder upsampled the features using 2 × 2 transposed convolutions, followed by two 3 × 3 convolutions and ReLU activations. A bottleneck of two 3 × 3 convolutions with ReLU connects the encoder and decoder, linking the high-level features to the upsampled output. The architecture of the U-Net is shown in Fig. 8.

### YOLO-v8

**Fig. 9:**
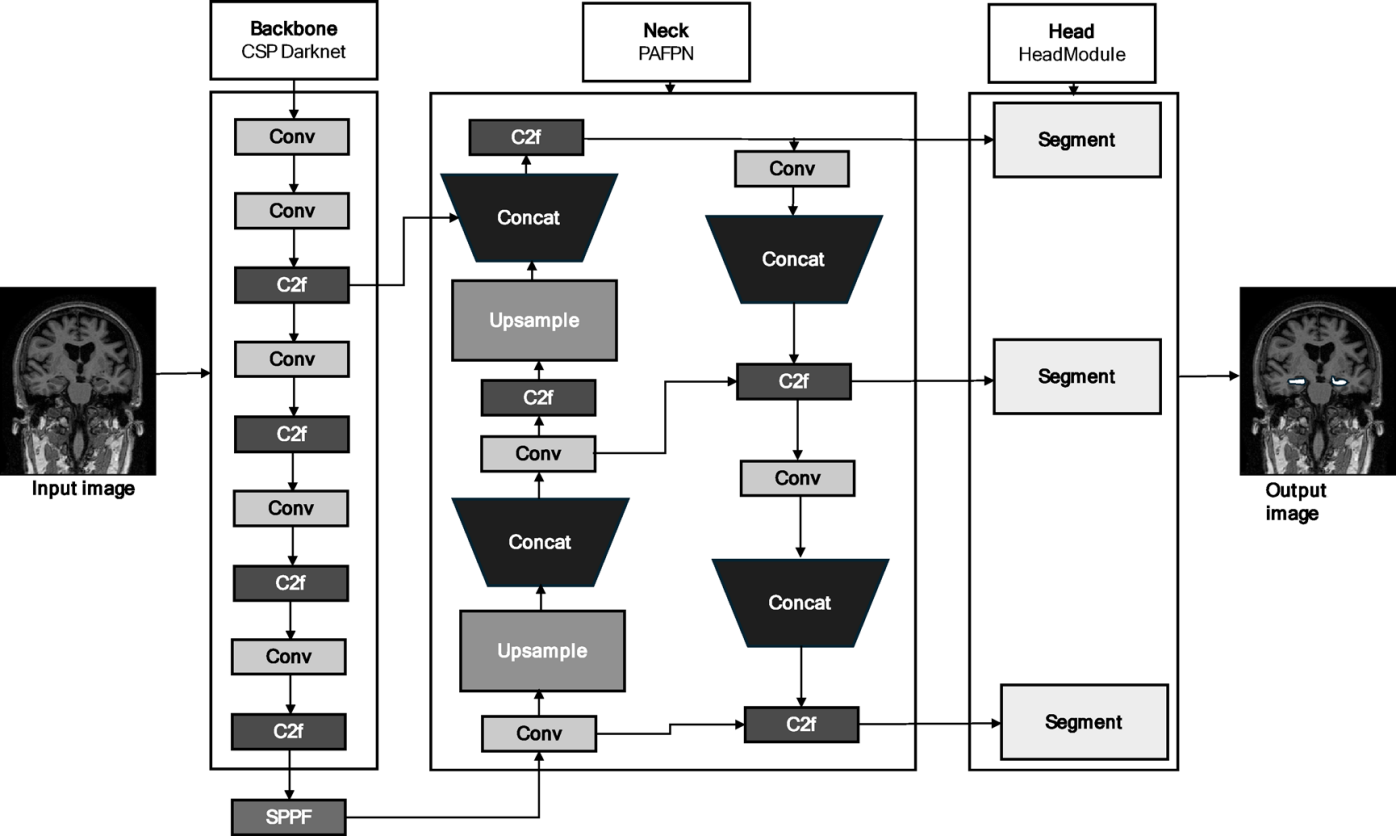
The YOLO-v8 architecture for hippocampus segmentation with sample MRI images sourced from the Alzheimer’s Disease Neuroimaging Initiative (ADNI) dataset.

The You Only Look Once (YOLO) series constitutes a deep learning paradigm designed for object detection. YOLO-v8, an evolution by the same authors as YOLO-v5, adheres to a comparable stylistic framework. Noteworthy advancements and optimizations characterize YOLO-v8, which surpasses the capabilities of the YOLO-v5 network and elevates the overall algorithmic performance. The YOLO-v8s-Seg model represents an extension of the YOLO-v8 object detection framework, which is explicitly tailored for executing segmentation tasks^55^. The architecture of the YOLO-v8 model is shown in Fig. 9.

### DeepLab-v3

**Fig. 10:**
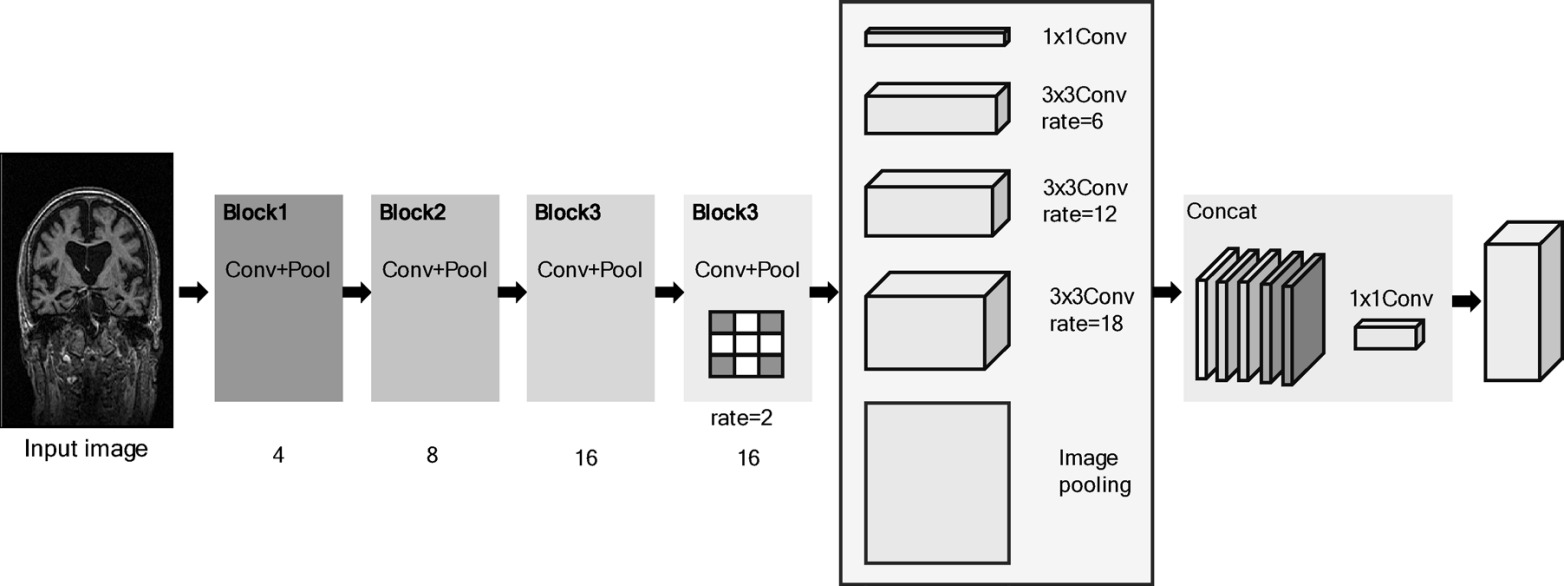
The DeepLab-v3 architecture (Conv stands for convolutional layer) with sample MRI images sourced from the Alzheimer’s Disease Neuroimaging Initiative (ADNI) dataset.

DeepLab-v3 is an advanced image-segmentation architecture. DeepLab-v3 aims to understand object boundaries better and make more accurate segmentations, particularly by using techniques such as atrous (dilated) convolution and Atrous Spatial Pyramid Pooling (ASPP)^56^. Additionally, MobileNet-v3 optimizes its network structure using a platform-aware Neural Architecture Search (NAS) approach. The model utilizes depth-wise separable convolutions, which significantly reduce the number of parameters and computational load, thereby enhancing its efficiency. Additionally, MobileNet-v3 incorporates squeeze-and-excitation modules and introduces a novel activation function, hard-swish, to further enhance performance while maintaining low resource consumption. Finally, DeepLab-v3 with MobileNet-v3 was established as the backbone. The architecture of the DeepLab-v3 model is shown in Fig. 10.

**Fig. 11:**
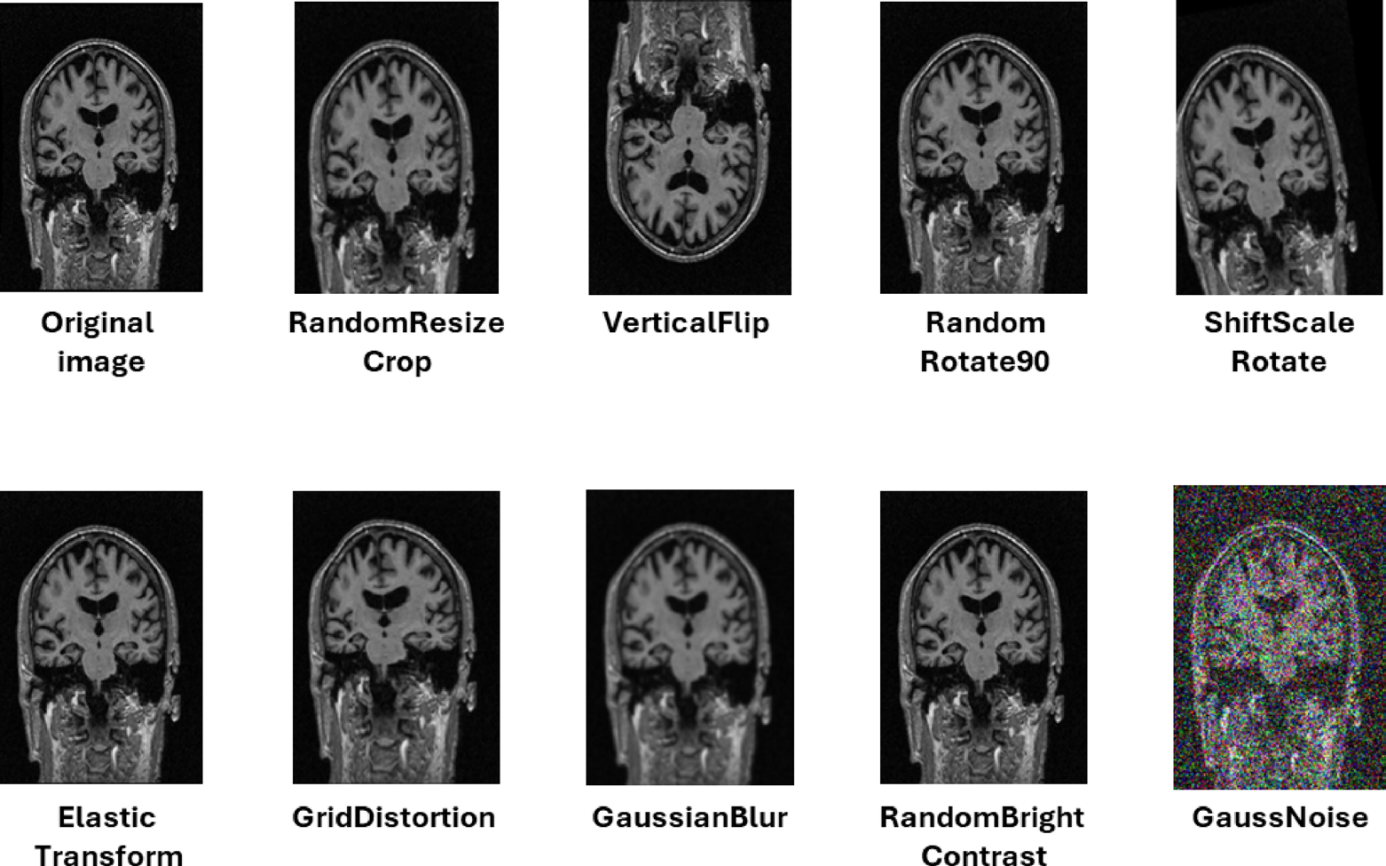
An example of data augmentation applied to the dataset with sample MRI images sourced from the Alzheimer’s disease neuroimaging initiative (ADNI) dataset.

In this study, we applied data augmentation because it was found to be the most widely used technique to expand the size of the training dataset, thereby improving deep learning performance. Following previous studies, our study implemented several data augmentation techniques^57^. We applied AutoAugment using the RandAugment implementation, where augmentation transformations were applied based on the settings of the experiment. We applied data augmentation to our proposed models. An overview of the data augmentation strategies implemented in U-Net, YOLO-v8, and DeepLabv3 is provided in Table 7; Fig. 11, which show the probability value of applying data augmentation to the dataset.

**Table 7:** Details of the data augmentation.

U-Net and DeepLab-v3 model	YOLO-v8 model
Random resized crop (scale = 0.8, 1.0)	Flip (*p* = 0.5),
Vertical flip (*p* = 0.5)	Hue shift augmentation (*p* = 0.015)
Random rotate90 (*p* = 0.5)	Saturation shift (*p* = 0.7)
Shift scale rotate (*p* = 0.8)	Value shift (*p* = 0.4)
Elastic transform (*p* = 0.5)	Translate (*p* = 0.1)
Grid distortion (*p* = 0.5)	Scale (*p* = 0.5)
Random brightness contrast (*p* = 0.5)	Crop fraction (*p* = 1.0)
Gauss noise (*p* = 0.5)	Erasing (*p* = 0.4)
Gaussian blur (*p* = 0.3)	Mosaic (*p* = 1.0)

*Note: p,* probability.

**Fig. 12:**
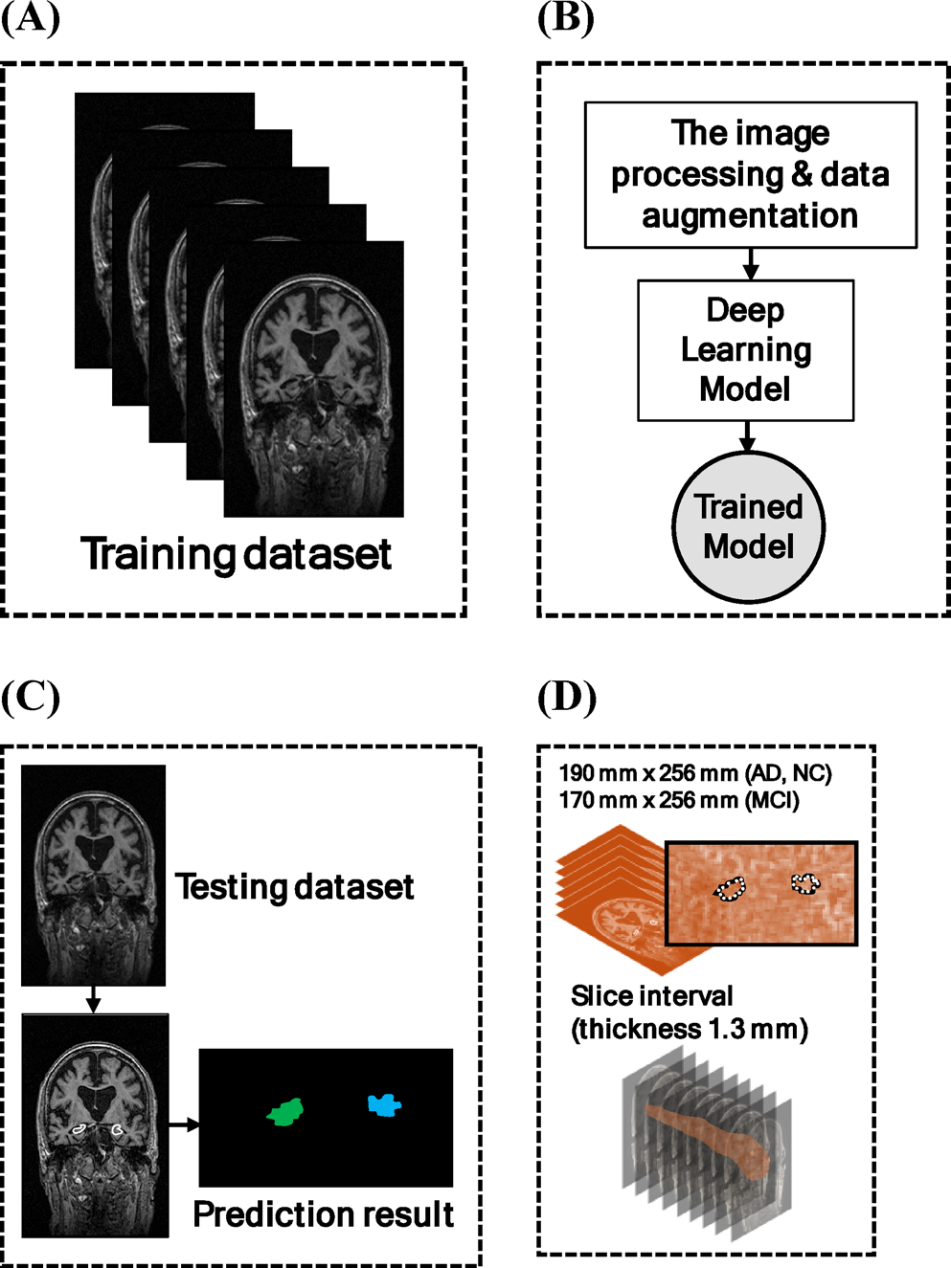
The illustration of the volume measurement of our study from sample MRI images sourced from the Alzheimer’s Disease Neuroimaging Initiative (ADNI) dataset; (**a**) The training dataset; (**b**) The image processing and data augmentation was applied to the dataset and was trained using deep learning, which used the best model performance; (**c**) The testing dataset was used to obtain the prediction result to get the segmentation masks and extract anatomical coordinates; and (**d**) The hippocampus volume measurement was measured with the slice interval in 1.3 mm thickness of each slice.

We used standard medical image segmentation evaluation metrics, such as the IoU, which was shown as one of the parameters due to its sensitivity to spatial overlap and its relevance for assessing the accuracy of segmenting small, complex anatomical structures. Additionally, we used precision and recall, which measure the ratio of correct predictions similarly. Furthermore, segmentation boundaries were generated using the model that exhibited the highest performance among the proposed models: U-Net, YOLO-v8, and DeepLab-V3. We selected the IoU as the parameter for optimal model performance. To assess the model’s generalization capability, we used data from another 30 subjects across three classes (i.e., AD (10 subjects), MCI (10 subjects), and NC (10 subjects)) comprising 814 image slices to generate segmentation masks and extract anatomical coordinates for subsequent analysis. An illustration of the hippocampus measurements is shown in Fig. 12**.** To verify the stability of the U-Net model, K-fold cross-validation was employed, which split our dataset into three subsets, meaning we trained the model *K* times, wherein a single partition was retained for training the model. The other was used for the validation data^58^. The use of three subsets is to learn robust features, particularly in medical image segmentation tasks where small structures such as the hippocampus are involved. This process was repeated three times to set the training set larger, which can ensure the model shows better performance, and each time, a different partition was used as validation data. All implementations and model training were performed in an Ubuntu 22.04-based environment with Python version 3.11.5 and ROCm 6.2 for AMD GPU support, running on a Giga Computing G493 Server with the following specifications: AMD EPYC 9654 96-Core processor, 755.5 GB RAM, and an AMD MI210 GPU with 64 GB of VRAM.

2 $${\mathrm{Volume}} = \sum\limits_{{i = 1}}^{{N - 1}} {(((L_{{P\:}} *X*Y)i*D))}$$

### Hippocampus volume measurement

Volume measurements were performed using the best segmentation performance obtained from the labeling coordinate data. The left and right hippocampus formation volumes were determined by coordinating with a 1.3 mm thickness in coronal views. We calculated the volumes using the results for each slice, interval information, and pixel size of the hippocampus (Eq. 2), which is consistent with previous studies^59^. An illustration of the volume measurements used in this study is shown in Fig. 12D.

where *N* is the number of slices, *i* is the slice number (approximately 25–30 slices in the coronal view), *D* is the slice interval (thickness 1.3 mm), *Lp* is the converter factor for the number of pixels (AD and NC class 190 mm × 256 mm; MCI class 170 mm × 256 mm), and *X* and *Y* are the coordinate pixels.

### Statistical analysis

The asymmetry index of the hippocampus volume was calculated by calculating the percentage ratio of the difference between the left and right hippocampus volumes (Eq. 3). If the value is the same as zero, there is symmetry between the left and right sides; if the value is greater than zero, the right side is larger than the left, following the previous studies^60,61^. The formula used is as follows:

3 $$Asymmetry\:Index=\frac{Right-Left}{Right+Left}*100$$

A paired *t* test was used to compare the left and right volumes separately for each class to explore the possible lateralization of the hippocampus volume. We excluded the statistical comparison for the metrics performance across models, since deep learning model performance depends on the split dataset^62^. Instead, we used K-fold cross-validation. One-way ANOVA with Fisher’s LSD post-hoc test was used to examine the efficacy of repeated measures between the AD, MCI, and NC groups. All statistical tests were performed using SPSS 22 (IBM, Somers, NY, USA) at a significance level of 0.05.

## Data Availability

The datasets used and/or analyzed during the current study were obtained from the Alzheimer’s Disease Neuroimaging Initiative (ADNI) database. Due to restrictions outlined in the ADNI Data Use Agreement, the datasets are not publicly available but can be accessed by qualified researchers upon request through the ADNI website: [https://adni.loni.usc.edu/](https:/adni.loni.usc.edu) . Furthermore, the corresponding source codes are publicly available through their respective official repositories on GitHub. First, the U-Net model: [https://github.com/milesial/Pytorch-UNet](https:/github.com/milesial/Pytorch-UNet) . Second, DeepLab-v3: [https://pytorch.org/hub/pytorch\_vision\_deeplabv3\_resnet101/](https:/pytorch.org/hub/pytorch_vision_deeplabv3_resnet101) . Last, YOLO-v8: [https://github.com/ultralytics/ultralytics](https:/github.com/ultralytics/ultralytics) .
